# A Dilemma: Electrographic Seizure Activity in the Absence of Clinically Perceptible Seizures and the Ethical Challenges of Medical Decision-Making

**DOI:** 10.7759/cureus.80331

**Published:** 2025-03-10

**Authors:** Thi Nguyen, Mirjana Ivanisevic, Anne Giles, Mohan Kurukumbi

**Affiliations:** 1 Neurology, University of Virginia School of Medicine, Charlottesville, USA; 2 Inova Neuroscience Institute, Inova Health System, Falls Church, USA

**Keywords:** case report, epilepsy, ethics, rns, seizures

## Abstract

A 37-year-old male with refractory left temporal epilepsy was admitted to the epilepsy monitoring unit to examine correlates of observable clinical seizure activity, those captured by responsive neurostimulation system (RNS) and continuous video electroencephalogram (cvEEG). The patient was diagnosed at age three, was on three anti-epileptic drugs, with an RNS implant since 2020 and was admitted to the epilepsy monitoring unit. The patient reported no seizures since 2019. cvEEG and RNS data were collected, and a comprehensive neuropsychological evaluation was conducted.

cvEEG revealed brief electrographic activity originating from the left and right anterior temporal regions, occurring mainly on the left side. The activity was characterized ictally by prominent anterior temporal sharp waves, with a left-sided predominance. RNS data showed similar results but recorded electrographic activity in excess of cvEEG. Although clinical and electrographic manifestations tend to be stereotyped for seizures, there were no behavioral observations of clinical seizures during these recorded electrographic seizures on RNS data. The patient also reported no seizures. Neuropsychological results showed impairment across multiple cognitive domains.

This case report highlights the need for a more detailed approach to determining allowable electrographic activity since these thresholds directly impact restrictions on patients with epilepsy. Highly sensitive measurement tools may better detect seizures, but in isolation, they cannot fully convey a complete picture of the patient’s status without other data and clinical indicators. Data from emerging technology must be weighed in conjunction with clinical symptoms to optimize patient safety, quality of life, and outcomes.

## Introduction

Epilepsy is a neurologic condition in which individuals experience involuntary, recurrent, and unprovoked epileptic seizures that involve the excessive normal firing of neurons, resulting in neurobiological, cognitive, and psychological changes [1]. This condition is more common in males compared to females and older individuals, as well as in developing or underdeveloped countries [2]. Epilepsy can be caused by many factors, including structural, genetic, infectious, metabolic, and immune [3]. There are two broad classifications for epilepsies: focal epilepsy and generalized epilepsy [4]. Focal impairment awareness epilepsy can be further categorized into seizure types, described by their respective features, for instance, motor or non-motor [5].

The incidence of epilepsy is estimated to be about 61.44 per 100,000 person-years, and the lifetime prevalence is 7.60 per 1,000 population [6]. Refractory epilepsy is defined as failure of seizure control despite trialing two or more anti-epileptic medications and affects 30 to 40% of individuals with epilepsy [7]. However, newer technology allows for the improved treatment of refractive epilepsy with the additional benefit of seizure detection for patients who have non-resectable lesions and an identified origin of seizure onset.

Seizures have been traditionally captured using electroencephalography (EEG). EEG consists of placing electrodes on the scalp to measure the electrical activity of the brain, with epileptiform activity consisting of excessive synchronous discharge of a population of neurons resulting in spikes, sharp waves, or spike-and-wave activity corresponding to seizures. This tool can also allow for the localization of seizure origin, with the temporal lobe being the most common [8]. EEGs allow for the objective detection of seizures and improved characterization. Although continuous EEG monitoring allows for the detection of subclinical seizures, the feasibility of prolonged continuous monitoring is limited. Patients can be admitted to the epilepsy monitoring unit, where they are continuously monitored, sometimes under video surveillance to confirm signs of clinical seizures and recognize seizure activity on cvEEG. The development of seizure detection algorithms assists with continuous monitoring, however, these methods are highly sensitive with uncertain clinical relevance at times. 

To date, the only FDA-approved continuous epilepsy monitoring device that provides feedback to patients and can send data to neurology physicians caring for patients is the NeuroPace responsive neurostimulation system (RNS) [9]. RNS consists of an implanted device under the scalp with electrodes positioned over identified areas of seizure origin. This technology monitors the electrical activity of the brain and also sends electrical signals to modulate this activity to reduce the frequency of seizures over time. RNS also sends additional electrical activity to stop seizures that have been detected in real-time. This tool is useful for individuals who have failed multiple seizure medications and/or wish to better monitor their seizure activity in real-time [10]. Eligibility criteria include standard candidacy for surgery and seizures occurring from a specific identifiable region in the brain with a maximum of 2 locations. The process of implantation involves the placement of a device under the scalp and 2 electrodes leading to those areas of seizure origin. Additional benefits include allowing for the identification of seizure triggers given patterns in brain activity and constant electrical stimulation from the device that allows for the treatment of seizures in real-time, leading to long-term neuromodulation similar to deep brain stimulation and vagus nerve stimulation but without associated side effects of depression and voice changes/coughing/gagging, respectively [11].

While this technology has allowed for the improved detection of seizures for more optimal treatment, here we describe a case in which the device has determined periods of electrographic activity in the brain in the absence of clinically observed perceptible seizures. To best optimize patient safety, it is important to recognize and reduce the frequency of seizures, and advanced detection tools may help with this process. The detection of these subclinical seizures in the absence of clinically observed seizures provides more detailed and precise information about seizure activity. However, challenges arise when best interpreting this data while also considering quality of life. The advancement of new technology also poses challenges for the clinical care team in the context of conflicting information gathering about ongoing seizures for patients, including, but not limited to, important decisions such as clearance for driving [12]. According to current guidelines in the state where our patient resides, driving is permissible if individuals have not experienced clinically perceptible seizures for the last six months. Ethical issues may stem from a lack of clarity in interpreting electrographic activity detected by highly sensitive newer technology such as RNS and attempting to determine whether these may be subclinical seizures. 

It is well known in the literature that advanced technology, including RNS, is highly sensitive, and physicians must be mindful of this when making medical decisions. However, there have not been previously published cases on how to manage similar situations to our knowledge, but we surmise that other practices may have similar approaches.

This case was previously presented as a poster at the 2023 American Epilepsy Meeting on December 2, 2023. By illustrating this case through the form of a paper, we hope to provide an example to highlight this dilemma and bring this issue to awareness regarding the need for more guidelines on how to best incorporate information obtained from this emerging technology into current clinical practice to optimize outcomes for patient safety and quality of life.

## Case presentation

**Figure 1:**
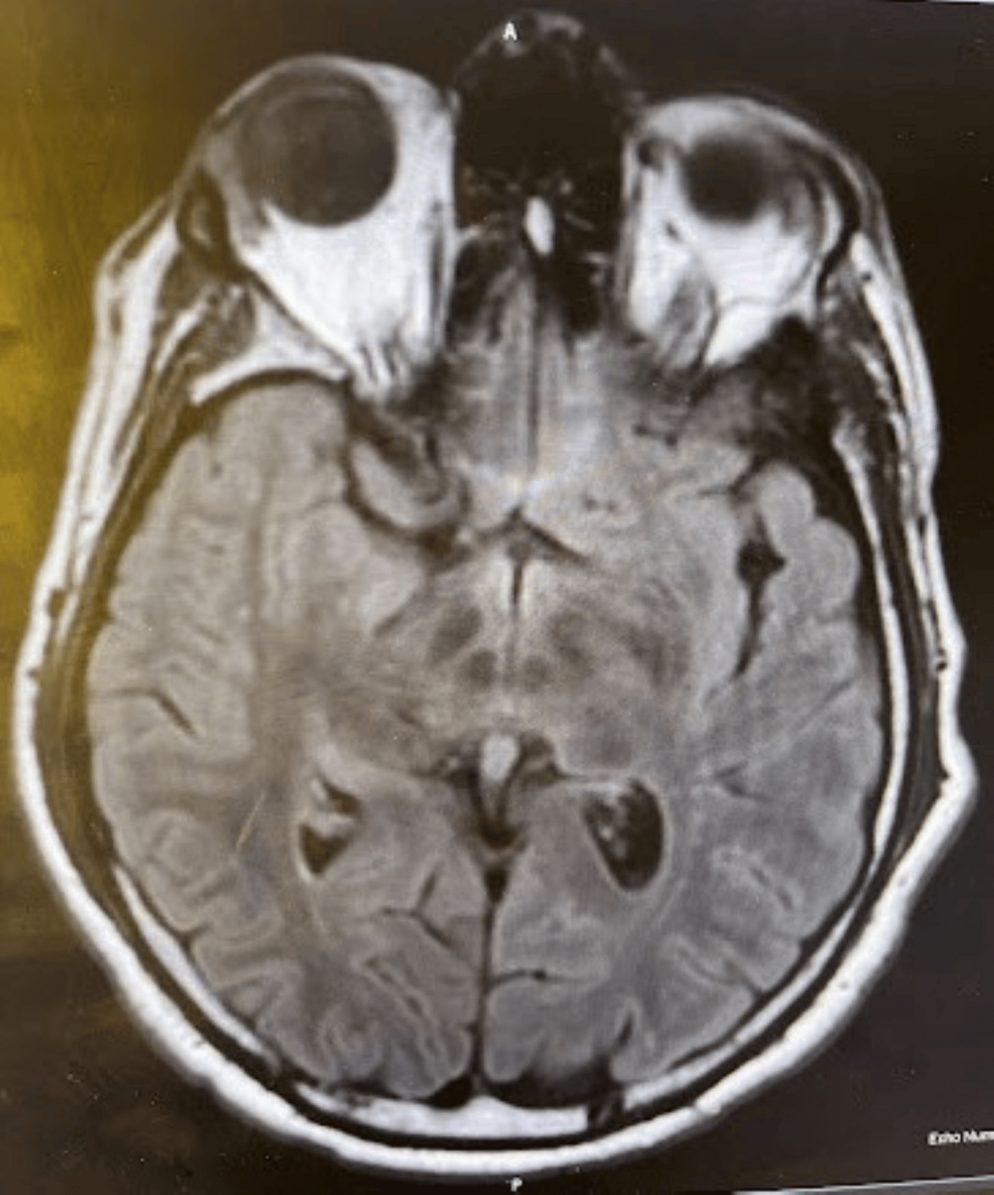
Magnetic resonance image (MRI) of the brain with and without contrast non-lesional for seizures.

In the present case presentation, we admitted a patient with epilepsy to monitor for any ongoing seizure activity post-RNS implantation. Our patient is a 37-year-old, right-hand dominant Caucasian male with refractory left temporal epilepsy with seizure onset of unclear etiology at the age of three. He had RNS implanted to control ongoing seizures. Magnetic resonance imaging (MRI) findings were unremarkable (Figure 1).

EEGs allow for the objective detection of seizures and improved characterization (Table 1).

**Table 1:** Chart illustrating advantages and disadvantages of seizure detection methods.

Technique	Description	Advantages	Disadvantages
Electroencephalography (EEG)	The traditional method of seizure capture consists of placing electrodes on the scalp to measure the electrical activity of the brain, allowing for seizure origin localization.	Allow for the objective detection, localization, and improved characterization of seizures, including subclinical ones.	Limited in the feasibility of prolonged continuous monitoring.
Epilepsy monitoring unit (EMU) Admission	Continuous monitoring for seizure signs and activity on continuous electroencephalography in the hospital for a prolonged period of time, often several days.	Similar to EEG, with the benefit of the monitoring of patients by experienced clinical staff. Admissions can also be used to determine optimal seizure medication regimens.	Requires hospital admission, which may not be feasible for patients.
Responsive neurostimulation system (RNS)	The implanted device under the scalp with electrodes positioned over identified areas of seizure origin. This technology monitors the electrical activity of the brain and also sends electrical signals to modulate this activity to reduce the frequency of seizures over time.	Continuous epilepsy monitoring device that provides feedback to patients and can send data to neurology physicians caring for patients. Also sends additional electrical activity to stop seizures that have been detected in real time. Useful for individuals who have failed multiple seizure medications and/or wish to better monitor their seizure activity.	May generate false positives where patients may not be experiencing any observable seizures. Eligibility criteria may limit certain patients from this option, including standard candidacy for surgery and seizures occurring from a specific identifiable region in the brain with a maximum of 2 locations.

**Figure 2:**
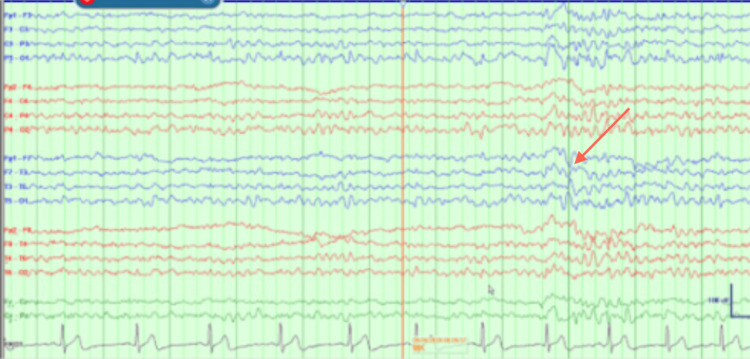
Wada results. Electroencephalography baseline prior to Wada procedure showing intermittent left temporal discharges.

**Figure 3:**
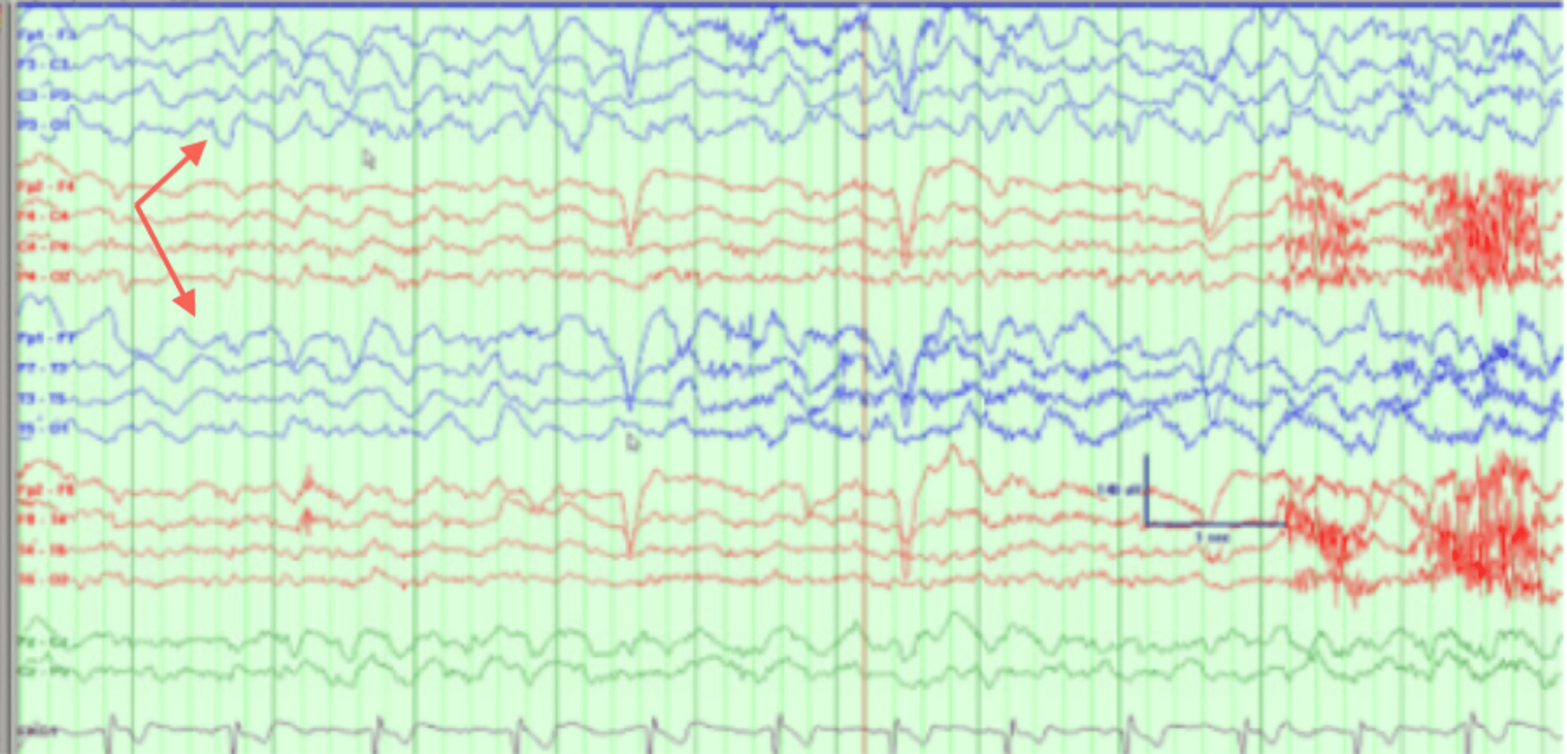
Wada results. Status post-injection on the left side. Electroencephalography shows focal slowing over the left hemisphere noted with shivering artifacts. No slowing was seen over the right side confirming no cross-over of the dye.

**Figure 4:**
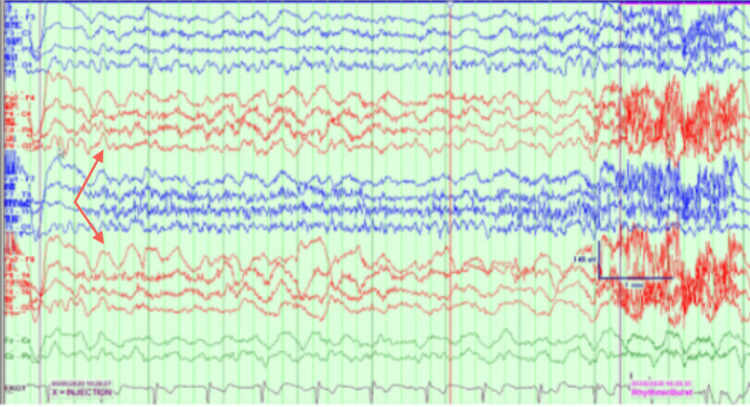
Wada results. Status post-injection on the right side. Electroencephalography shows focal slowing over the right hemisphere.

EEG findings showed multiple seizures originating from a left temporal onset. A positron emission tomography (PET) scan showed left temporal hypometabolism. Wada testing from May 2020 revealed 8/8 left memory, 4/8 right memory, and language localized to the left hemisphere (Figures 2-4).

His medical history is unremarkable, and his psychiatric history is significant for depression. He was currently taking the following medications for managing seizures: levetiracetam, lamotrigine, and lacosamide in optimal doses. His review of systems was pertinent for a history of seizures. He was well-developed and in no acute distress. His physical exam was within normal limits. His mental status was within normal limits, and he was awake, alert, and oriented to person, place, and time. Overall, there were no focal deficits on examination. His seizure activity was monitored with multiple modalities, including cvEEG during his epilepsy monitoring unit admission and continuous RNS data findings.

cvElectroencephalography (cvEEG) and responsive neurostimulation (RNS) data findings

**Figure 5:**
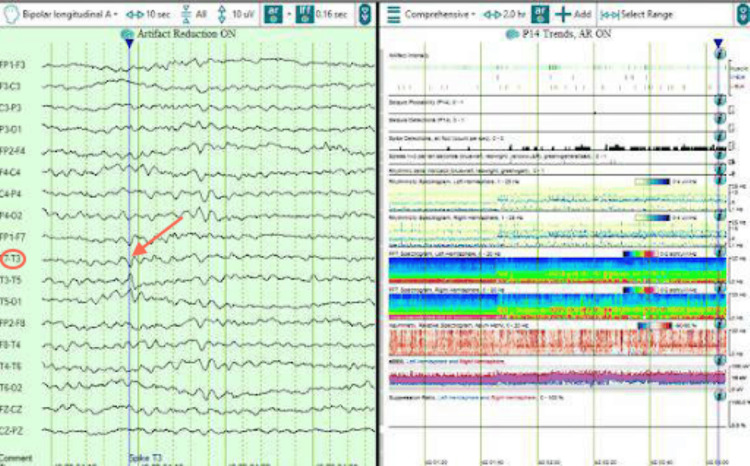
Interictal left anterior temporal F7T3 sharp wave.

**Figure 6:**
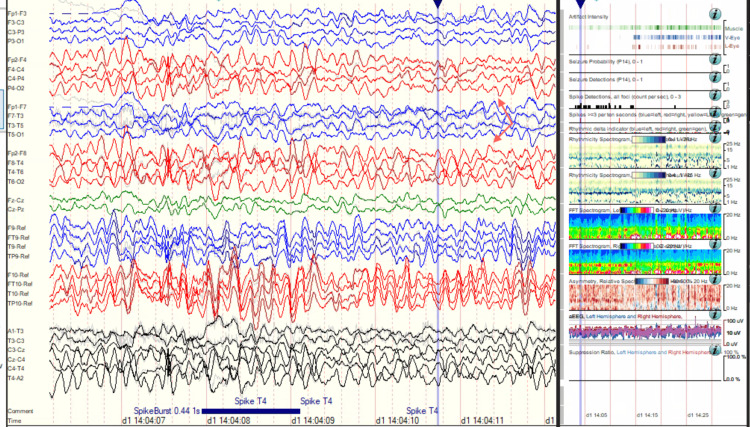
Interictal bilateral with mid-temporal rhythmic theta slowing, more marked on the right than left.

**Figure 7:**
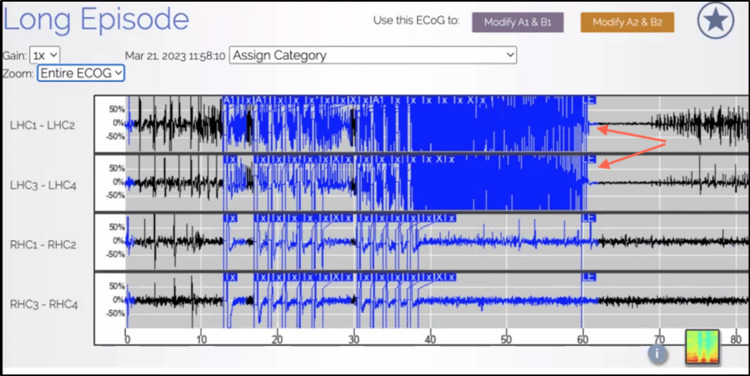
Responsive neurostimulation system (RNS)/electrocorticography (ECoG) demonstrating electrocorticogram activity originating from left hippocampal onset in a 60-second snapshot.

During this current admission, interictal cvEEG showed prominent anterior temporal sharp waves, with a left-sided predominance (Figure 5), and showed bilateral anterior/mid-temporal rhythmic theta greater than delta activity, more marked on the right than the left (Figure 6). The RNS data showed similar results but recorded more electrographic activity compared to cvEEG findings (Figure 7). There were no behavioral observations during this recorded RNS electrographic seizure activity. The patient also reported no seizures.

Neuropsychological results

Results from his neuropsychological evaluation demonstrated impaired performance across multiple domains, including processing speed, visual and verbal learning and memory, language, and some aspects of executive functions. The impairment in his executive function skills was especially apparent in cognitive flexibility. While his verbal reasoning skills, conceptual reasoning skills, working memory, attention, and visuospatial skills were intact, he presented with weakness in his executive function skills, specifically in psychomotor task switching (Table 2). 

**Table 2:** Neurocognitive profile summary. *Impaired on cognitive flexibility. Weakness in psychomotor task switching. “X” means that the category listed applies to the patient.

Domain	Intact	Weakness	Impaired
Verbal Reasoning Skills	X	-	-
Conceptual Reasoning Skills	X	-	-
Working Memory	X	-	-
Processing Speed	-	-	X
Attention	X	-	-
Visual Learning	-	-	X
Visual Memory	-	-	X
Verbal Learning	-	-	X
Verbal Memory	-	-	X
Language	-	-	X
Executive Function Skills*	-	X	X
Visuospatial Skills	X	-	-

## Discussion

Our patient experienced uncontrolled seizures secondary to a strong left temporal dominance. Several trials of multiple medications did not control his seizures, and furthermore, because his seizures could not be precisely localized, he was not a strong candidate for surgical intervention options. It is for patients with lesional epilepsy that could not be resected, such as him, that new technology, such as RNS, offers an ideal opportunity for seizure control [13-14].

We admitted our patient with refractory epilepsy to the epilepsy monitoring unit post-RNS implantation to monitor ongoing seizure activity and guide clinical decision-making for his quality of life. cvEEG showed brief electrographic seizures, occurring mainly on the left temporal side. RNS data showed similar results but demonstrated electrographic activity in excess of the cvEEG findings. Neuropsych data revealed impaired performance across multiple domains. However, our patient did not report experiencing clinical seizures during his admission to the epilepsy monitoring unit.

Our findings suggest that the patient is not experiencing disabling seizures that he is aware of, and this was supported by cvEEG findings from the inpatient stay. However, neuropsychological findings reveal cognitive deficits, especially in the processing speed. This, coupled with ongoing electrographic seizure activity that was captured by RNS, presents challenges for the clinical care team when making decisions about the patient's ability to drive and quality of life. While RNS technology has improved the detection of seizure activity, there arises a question of optimally managing the absence of seizure-related symptoms with conflicting electrographic recordings. This poses a challenge to the clinical care team regarding how to best incorporate this data into medical decision-making. The limitations described by the patient concerning his driving restrictions manifest as depression and anxiety, ultimately interfering with his quality of life [15-16].

Even after epilepsy monitoring unit admission, seizure activity has continued to be observed with the RNS data, especially with napping, and medication changes have been made accordingly. Since RNS data was in excess of cvEEG used to characterize subclinical seizures and without observable symptoms, we have determined that it may not be as clinically relevant in this particular case, given the propensity of highly sensitive newer technology. On follow-up visits, we have continually been working towards optimal medication doses to minimize side effects while effectively reducing seizure frequency. He is currently managed on lacosamide, lamotrigine, levetiracetam, midazolam as needed, cannabidiol, and cenobamate on optimal doses. His review of systems and physical exam have remained unchanged. He notes regular medication compliance and is doing well overall. We also discussed the risks and benefits of driving and mutually agreed upon a 25-mile radius after patient acknowledgment and agreement to go to the local driving rehabilitation center to aid in this process. He has since been able to resume work and drive to the grocery store in the past months since his epilepsy monitoring unit admission and driving rehabilitation sessions. Driving, which allowed our patient to resume work, was very important to him regarding his view on the clinical situation. He reported being pleased with his increased ability to drive to work in his rural town and working on a more consistent routine with increased regular work hours despite his condition. Overall, his perceived quality of life was improved. 

Strengths of this case report include that it incorporates the use of newer technology and focuses not only on effective treatment as a goal but also on quality of life considerations for our patient. Limitations include the fact that this is one patient and that the lessons learned from this case may not be completely generalizable to the rest of the population, especially with regard to states with different regulations on driving restrictions and epilepsy. However, this patient does represent issues that may manifest in others, which will need to be addressed on a case-by-case basis.

The new evidence provided by more sensitive measurement tools only further adds to the need for ongoing work into medical treatment for seizures to potentially redefine what constitutes a seizure. This issue is important not only for this particular patient but also for other patients in similar situations who wish to expand their access to privileges afforded by a lack of seizures [17-20]. We propose that newer guidelines are needed for clinical decision-making that would assist providers with informing patients about the risks and benefits of this emerging technology to manage seizures in the context of both safety and quality of life.

**Figure 8:**
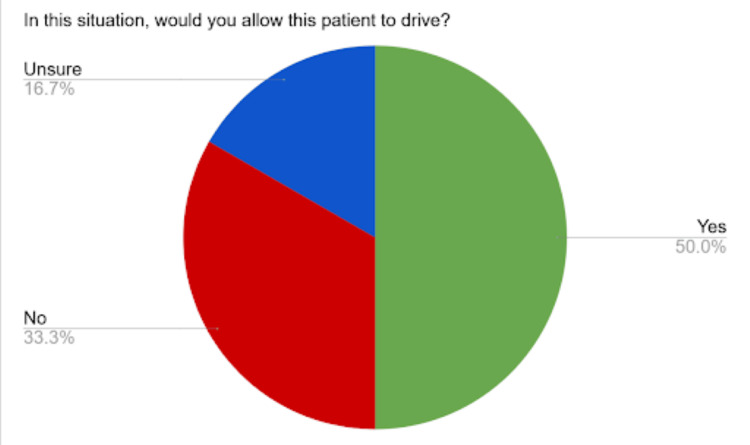
American Epilepsy Society (AES) survey results.

This case report was presented at the 2023 American Epilepsy Society (AES) national meeting. We polled our audience with a one-question survey asking them if they would allow our patient to drive considering his clinical presentation. At the conference, we noted that there was a wide net of providers who were unsure about how to handle a situation similar to what has been outlined in our case report. According to the results of the survey, 50% of participants responded yes, 33.3% no, and 16.7% unsure or need to gather more information (Figure 8). Some of the rationale provided included a reference to state laws, objective data demonstrated by the cvEEG and RNS data, and consideration for the patient’s overall functionality and well-being. Some audience members relied heavily on the clinical data provided, while others cautioned against the use of too much information clouding medical judgment. Others were comfortable with allowing the patient to drive based on empirical evidence while counseling him to be cautious with nocturnal driving given the association with subclinical activity occurring near episodes of transition to wakefulness from sleeping. Although the sample size was small, this demonstrates the complex nature of our case, and our poster presentation served as the basis for enriching discussions on medical ethics. We hope to continue these conversations with the publication of this case report.

## Conclusions

Overall, it is clear that recurrent seizures influence the quality of life in patients with epilepsy. Lifestyle measures, medications, and technology to help mitigate seizures can be employed as methods to mitigate this burden on patients. Providers caring for patients outside of the epilepsy monitoring unit have traditionally relied on the presence of clinical symptoms to detect seizures. While more advanced technology to improve the monitoring of seizures helps healthcare teams better detect seizures, it is also important to consider the electrographic data with regard to the clinical context. While it may be challenging to determine an appropriate threshold for measured electrical activity when placing restrictions on patients with epilepsy, this case report aims to call this matter to attention so that further research may be conducted to better define what constitutes seizure activity and provide improved clarity and outcomes and a better quality of life for patients as a whole.

## References

[1] Stafstrom CE, Carmant L (2015). Seizures and epilepsy: an overview for neuroscientists. Cold Spring Harb Perspect Med.

[2] Nayak CS, Bandyopadhyay S (2024). Mesial Temporal Lobe Epilepsy. https://pubmed.ncbi.nlm.nih.gov/32119319/.

[3] Christian E, Christian H (2018). Diagnostic challenges in epilepsy: seizure under-reporting and seizure detection. Lancet.

[4] Guery D, Rheims S (2025). Clinical management of drug resistant epilepsy: a review on current strategies. Neuropsychiatr Dis Treat.

[5] Fisher RS, Cross JH, French JA (2017). Operational classification of seizure types by the International League Against Epilepsy: position paper of the ILAE commission for classification and terminology. Epilepsia.

[6] Fiest KM, Sauro KM, Wiebe S (2017). Prevalence and incidence of epilepsy: a systematic review and meta-analysis of international studies. Neurology.

[7] Engel J Jr (2014). Approaches to refractory epilepsy. Ann Indian Acad Neurol.

[8] Thomas GP, Jobst BC (2015). Critical review of the responsive neurostimulator system for epilepsy. Med Devices (Auckl).

[9] The RNS System (2025). The RNS system. https://www.neuropace.com/patients/neuropace-rns-system/.

[10] Vera-González A (2022). Pathophysiological mechanisms underlying the etiologies of seizures and epilepsy. Epilepsy [Internet].

[11] Beghi E (2020). The epidemiology of epilepsy. Neuroepidemiology.

[12] Yang L, Wang Y, Chen X, Zhang C, Chen J, Cheng H, Zhang L (2021). Risk factors for epilepsy: a national cross-sectional study from national health and nutrition examination survey 2013 to 2018. Int J Gen Med.

[13] Hegde M, Chiong W, Rao VR (2021). New ethical and clinical challenges in "Closed-loop" neuromodulation. Neurology.

[14] Bergey GK, Morrell MJ, Mizrahi EM (2015). Long-term treatment with responsive brain stimulation in adults with refractory partial seizures. Neurology.

[15] Nair DR, Laxer KD, Weber PB (2020). Nine-year prospective efficacy and safety of brain-responsive neurostimulation for focal epilepsy. Neurology.

[16] Krishnan V (2020). Depression and anxiety in the epilepsies: from bench to bedside. Curr Neurol Neurosci Rep.

[17] Seid Jemal and Kalayu Mebrahtu (2022). Prevalence and associated factors of depression among people with epilepsy in Ethiopia: a cross-sectional study. Egypt J Neurol Psychiatr Neurosurg.

[18] Nigussie K, Lemma A, Sertsu A, Asfaw H, Kerebih H, Abdeta T (2021). Depression, anxiety and associated factors among people with epilepsy and attending outpatient treatment at primary public hospitals in northwest Ethiopia: A multicenter cross-sectional study. PLoS One.

[19] Joshi CN, Vossler DG, Spanaki M, Draszowki JF, Towne AR (2019). Chance takers are accident makers: are patients with epilepsy really taking a chance when they drive?. Epilepsy Curr.

[20] Krumholz A (2009). Driving issues in epilepsy: past, present, and future. Epilepsy Curr.

